# Pulmonary Embolism in a Patient With Undifferentiated Dyspnoea

**DOI:** 10.7759/cureus.94837

**Published:** 2025-10-18

**Authors:** Yun Hung Chor

**Affiliations:** 1 Emergency Department, Heartlands Hospital, Birmingham, GBR

**Keywords:** acute pulmonary embolism, dyspnoea of unknown origin, intravenous thrombolysis, massive pulmonary embolism, pulmonary oedema

## Abstract

Pulmonary embolism can cause right and left ventricular dysfunction and induce acute heart failure or shock. A 76-year-old gentleman with a history of ischaemic heart disease and atrial fibrillation, on oral warfarin and clopidogrel daily, presented with a three-day history of worsening shortness of breath associated with a dull ache over the right side of his chest. On examination, auscultation of his lungs revealed mild crepitations bilaterally at the bases and bilateral pitting oedema of legs up to his shins. His ECG showed ST-segment depression in lead 1, aVL, V2 to V6. His Chest X-ray showed cardiomegaly with minimal bilateral lower zone haziness. The initial provisional diagnosis was decompensated heart failure, and the patient was referred to acute medicine. His blood tests later showed a white cell count (WCC) of 15, CRP 298 and D-Dimer of 35317. This was followed by a computed tomography (CT) pulmonary angiogram, which showed multiple bilateral proximal pulmonary emboli with right heart strain.

A massive pulmonary embolism can present with non-specific signs and symptoms, which makes it difficult to diagnose. Clinicians should maintain a high index of suspicion as a patient on blood thinner cannot be excluded from having a pulmonary embolism. Acute heart failure and pulmonary embolism can clinically present in a similar way and often share similar risk factors. The patient developed a pulmonary embolism despite being on both warfarin (anticoagulation) and clopidogrel (antiplatelet).

Even with the latest advanced therapies and anticoagulation therapies, mortality remains high. Patients who have received a direct-acting oral anticoagulant (DOAC) will have significantly lower rates of progression to a pulmonary embolism than those on warfarin. Many factors can affect the therapeutic effect of warfarin. This case showed that pulmonary embolism can occur despite dual anticoagulant and antiplatelet therapy, particularly when therapy is subtherapeutic. It also highlights the importance of maintaining high clinical suspicion for pulmonary embolism even in patients who are taking anticoagulation therapy or dual anticoagulation and antiplatelet therapy. It suggests that DOACs may provide more reliable anticoagulation than warfarin in selected patients.

## Introduction

The epidemiology of pulmonary embolism (PE) can be difficult to predict, as the first presentation of a patient can be cardiac arrest. We present a case of undifferentiated dyspnoea with heart failure, which was caused by PE along with pre-existing cardiac disease. This patient developed a PE despite receiving dual therapy with warfarin and clopidogrel. We also highlight the importance of factors affecting the efficacy of vitamin K antagonists and the effectiveness of direct-acting oral anticoagulants (DOAC).

Acute heart failure and PE can clinically present in a similar way and often share similar risk factors. The diagnosis of dyspnoea can be difficult. Hypertension, dyslipidemia, diabetes, obesity, smoking, malnutrition, stress and estrogen therapy have significant effects on endothelial function, inflammation and hypercoagulable state. This results in the development of atherothrombosis, which leads to ischaemic heart disease, heart failure, and venous thrombosis. These factors can ultimately cause PE. Patients with cardiac disease, such as coronary artery disease and atrial fibrillation, also display a higher risk for PE. Many studies have shown that patients with previous myocardial infarction have a high risk of developing PE. Patients with heart failure have nearly double the risk of developing PE than those without it [1]. In addition, PE can cause right and left ventricular dysfunction and induce acute heart failure or shock. The diagnosis of massive PE is difficult because of its non-specific clinical presentation and the absence of definitive findings on non-invasive cardiac imaging. In around one third of patients, a massive PE presents with pleuritic chest pain with or without dyspnoea. The patient may present with retrosternal chest pain that mimics angina. Besides, cardiac syncope can be a hallmark of massive PE with ventricular failure and severe pulmonary hypertension. Even with the latest advanced therapies and anticoagulation therapies, mortality remains high [2]. This case illustrates a diagnostic challenge, describing a patient who presented with undifferentiated dyspnoea and was found to have a massive PE despite ongoing dual anticoagulation and antiplatelet therapy thereby highlighting the complex diagnostic and therapeutic challenges involved.

## Case presentation

A 76-year-old gentleman with a history of ischaemic heart disease and atrial fibrillation, and on oral warfarin 5 mg and clopidogrel 75 mg daily, presented with a three-day history of worsening shortness of breath associated with a dull ache over the right side of his chest. He had also developed bilateral leg swelling and orthopnoea. He had reduced mobility for two days due to shortness of breath and claimed that he had been compliant with his medication. He had last taken his dose of clopidogrel and warfarin the evening before he came to the hospital. There were no symptoms of fever or coughing. Prior to this presentation, he was fully independent in his activities of daily living and did not use mobility aids. He denied any recent medication or dietary change. We were unable to retrieve his recent INR readings from the anticoagulation clinic prior to admission; however, he claimed the recent reading was optimal.

**Figure 1:**
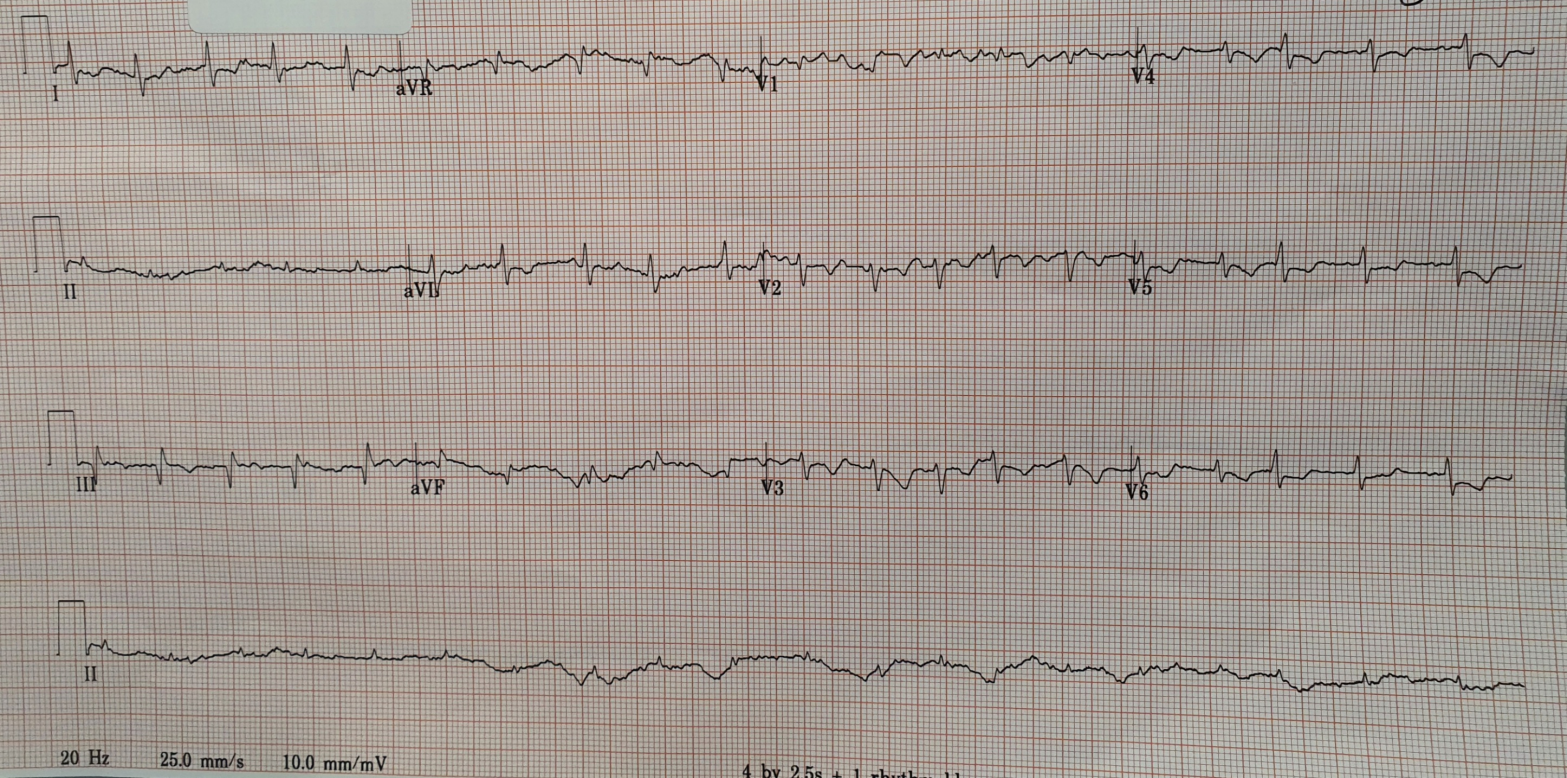
ECG of the patient

On arrival, his oxygen saturation on room air was 90%, blood pressure was 85/60 mmHg, temperature was 37.5 °C, heart rate was between 90-120 bpm, and Glasgow coma score was 15/15. On examination, auscultation of his lungs revealed mild crepitations bilaterally at the bases and bilateral pitting oedema of legs up to his shins. His ECG showed an ST-segment depression in lead 1, aVL, V2 to V6 (Figure 1).

**Figure 2:**
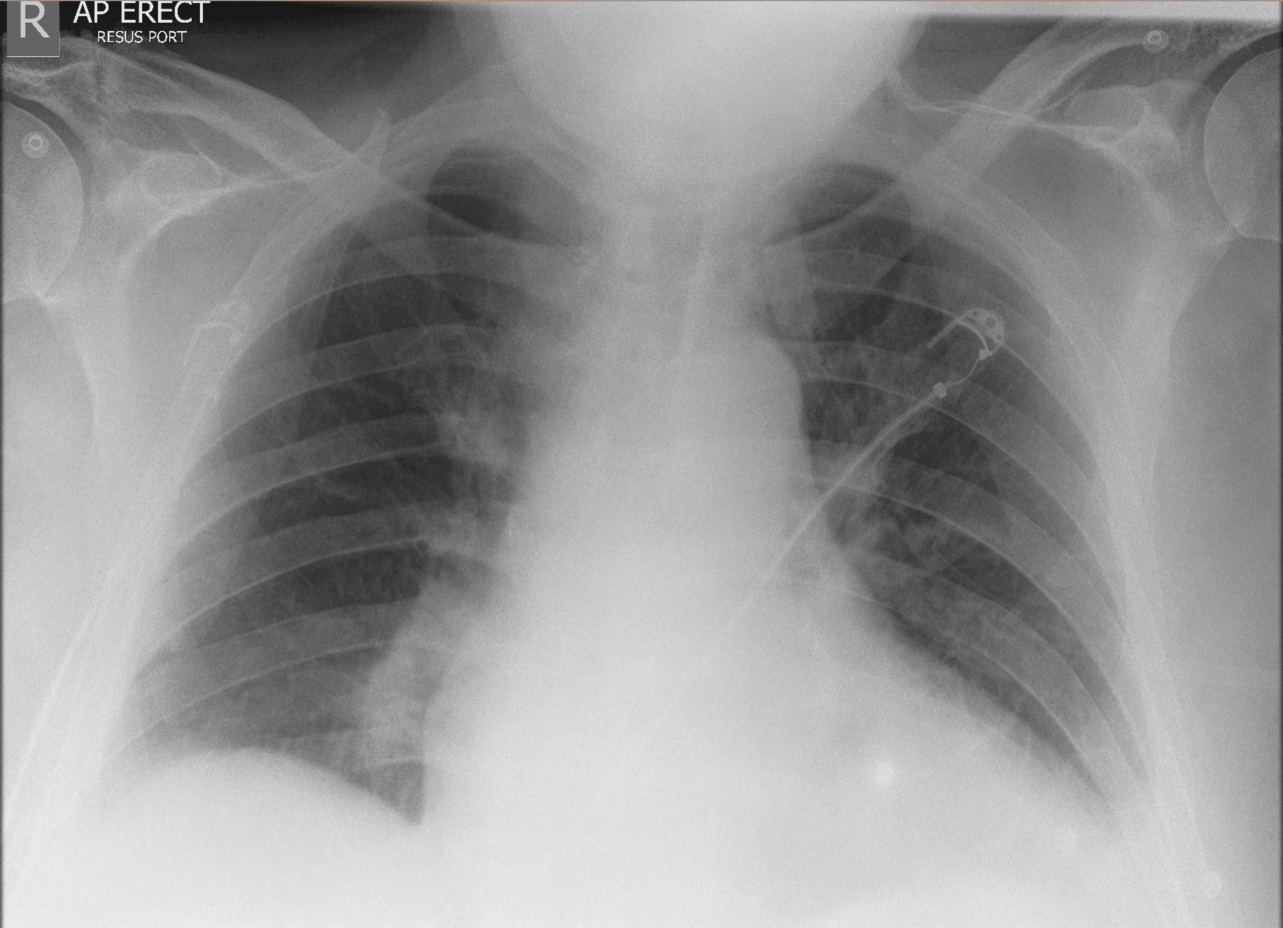
Chest X-ray of the patient

His arterial blood gas showed type 1 respiratory failure, and his chest X-ray showed cardiomegaly with minimal bilateral lower zone haziness (Figure 2).

He had a Wells' score [2] for PE of 1.5. The Initial provisional diagnosis was decompensated heart failure, and the patient was referred to acute medicine. There was ongoing concern that this could be related to an acute coronary syndrome, and Cardiology was also involved. He was given a total of 1 litre of intravenous fluid, in incremental boluses of 250ml; however, his blood pressure did not improve. His blood tests (Table 1) showed high values of D-dimer, white cell count (WCC), and C-reactive protein (CRP).

**Table 1:** Laboratory investigations

Parameter	Patient's values	Reference values
White cell count (WCC; x10^9 ^/L	15 (neutrophils 12.19)	4.0-11.0
Haemoglobin (Hb; g/L)	168	130-180
Platelet (g/L)	190	150-450
D-dimer (ng/ml FEU)	35317	0-500
C-reactive protein (CRP; mg/L)	298	<5
Bilirubin (μmol/L)	44	0-21
Alkaline phosphatase (ALP; U/L)	263	30-130
Total protein (g/L)	73	60-80
Alanine aminotransferase (ALT; U/L)	53	10-50
Troponin T (ng/L)	39	<14
B-type natriuretic peptide (NT-pro BNP; pg/ml)	5242	<400
International normalised ratio (INR)	1.5	1.0-1.3
Partial thromboplastin time ratio (PTR)	0.8	0.9-1.1
Urea (mmol/L)	9.3	2.5-7.8
Sodium (mmol/L)	138	133-146
Potassium (mmol/L)	4.7	3.5-5.3
Creatinine (μmol/L)	134	59-104
COVID and Influenza polymerase chain reaction (PCR)	Negative	

**Figure 3:**
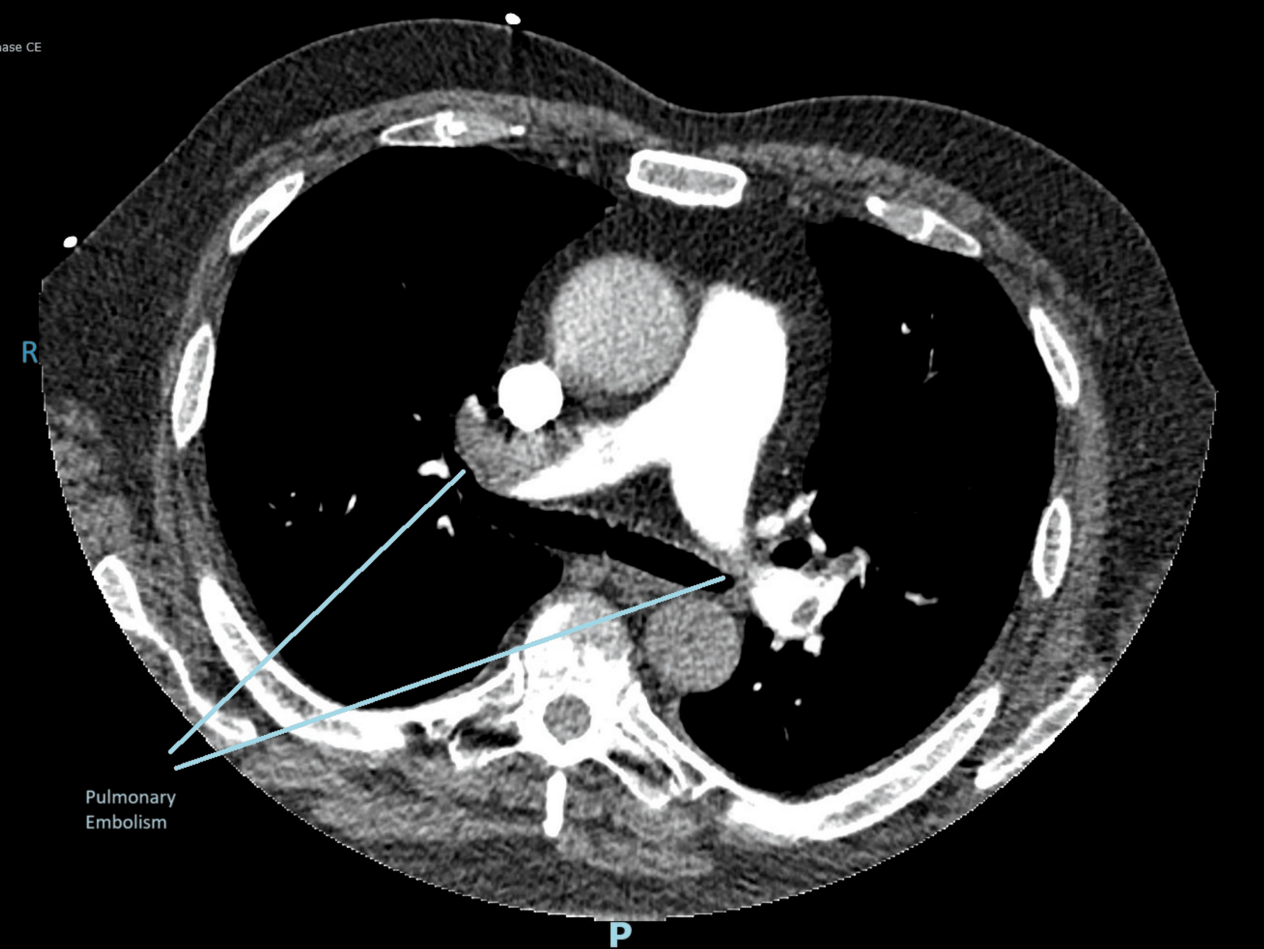
CT pulmonary angiogram

Following the D-dimer result, patient underwent a computed tomography (CT) pulmonary angiogram. It showed multiple bilateral proximal pulmonary emboli with right heart strain. The pulmonary infarcts in the middle lobe and right lower lobe may explain the right upper quadrant pain (Figure 3).

He was referred to the respiratory and intensive care teams and underwent thrombolysis in critical care setting. He was discharged home with DOAC after five days of hospital admission. No echocardiogram was performed during admission.

## Discussion

Nearly 45% of patients with PE will have a right ventricular compromise. There is a reported mortality of up to 25% in a normotensive patient and up to 65% in hypotensive patients with PE.. Massive PE occurs in about 5% of patients with PE and carries a mortality rate of 18-65% [3]. PE can present with non-specific signs and symptoms, making it a challenge to diagnose clinically. It can overlap with heart failure symptoms, especially in patients with underlying ischaemic heart disease.

A key learning point in this case was the development of PE despite the patient being on both warfarin (anticoagulation) and clopidogrel (antiplatelet) treatment. Patients who have received a DOAC will have significantly lower rates of progression to PE and a lower incidence of major bleeding compared with patients receiving warfarin. DOAC also has more predictable anticoagulation. This suggests that DOACs are superior to warfarin for the treatment of deep vein thrombosis [4].

However, it is important to note that DOACs have a relatively short duration of action; therefore, missing even a single dose can result in a rapid loss of anticoagulant effect. This makes the patient's compliance with the medication especially important compared to warfarin, especially after 12 to 24 hours. With warfarin, some benefit is retained for 48 to 72 hours after missing a dose, as it has a longer duration of action [5].

This case also highlights the real-world limitations of vitamin K antagonists, particularly when INR is subtherapeutic. Warfarin can also be affected by other factors. For instance, eating an increased amount of foods rich in vitamin K can lower the prothrombin time (PT) and INR, making warfarin less effective and potentially increasing the risk of blood clots [6]. Some medications can affect warfarin efficacy. An enzyme CYP P450 inducer can lead to a reduction in INR. Some drugs, such as rifampicin and phenytoin, that are enzyme inducers may lower the INR. Chronic alcohol consumption can also have an impact [7].

A D-dimer test is a sensitive but poorly specific diagnostic tool for a patient suspected with a PE (sensitivity 100%, specificity 8.8%). It should, therefore, always be used in conjunction with clinical suspicion [8]. The effect of DOACs and vitamin K antagonists such as warfarin on D-dimer levels was studied previously in patients with atrial fibrillation with inconsistent results. One of the studies showed that D-dimer levels in patients with atrial fibrillation on apixaban were increased in comparison to warfarin users [9].

Dual therapy (oral anticoagulant plus a single antiplatelet agent) has been shown to be effective in reducing thrombotic risks for one year in patients with cardiovascular conditions, including coronary artery disease, atrial fibrillation, and venous thromboembolism. This is particularly noticed when DOACs are combined with the antiplatelet, clopidogrel. Aspirin also reduces risk of cardiovascular death, myocardial infarction, stroke and stent thrombosis particularly in the first 30 days but the benefit does not extend beyond that time point [10].

This case shows the importance of maintaining a high clinical suspicion for PE, even in patients whose presentation mimics heart failure or acute coronary syndrome. Early diagnosis and treatment are the key to survival for such patients, especially when they present with a massive PE, as in our case. This case also supports the clinical transition of warfarin to DOAC especially in patients with complicated ischaemic heart disease and those with subtherapeutic INR. 

## Conclusions

PE presents with many symptoms and risk factors, which are often similar to other acute coronary conditions and acute heart failure, making early diagnosis very difficult. Clinicians will need to have a high level of suspicion even when the patient is on anticoagulation, or as in this case, where the patient was on both anticoagulation and antiplatelet therapy. PE can still happen, particularly when therapy is subtherapeutic or even when anticoagulation is combined with an antiplatelet agent. Additionally, DOACs may provide more reliable anticoagulation than warfarin in selected patients requiring both anticoagulant and antiplatelet therapy, as warfarin can interact with other medications and food. We need to have individualised anticoagulation strategies for different patients, including consideration of DOACs, which may improve outcomes and reduce the risk of thromboembolism in patients with complex cardiovascular conditions.
